# Role of Clock Genes and Circadian Rhythm in Renal Cell Carcinoma: Recent Evidence and Therapeutic Consequences

**DOI:** 10.3390/cancers15020408

**Published:** 2023-01-07

**Authors:** Matteo Santoni, Javier Molina-Cerrillo, Giorgio Santoni, Elaine T. Lam, Francesco Massari, Veronica Mollica, Giulia Mazzaschi, Bernardo L. Rapoport, Enrique Grande, Sebastiano Buti

**Affiliations:** 1 Oncology Unit, Macerata Hospital, Via Santa Lucia 2, 62100 Macerata, Italy; 2 Department of Medical Oncology, Hospital Ramón y Cajal, 28029 Madrid, Spain; 3 Scuola di Scienze del Farmaco e dei Prodotti della Salute, Università di Camerino, 62032 Camerino, Italy; 4 University of Colorado Anschutz Medical Campus, Aurora, CO 80045, USA; 5 Medical Oncology, IRCCS Azienda Ospedaliero-Universitaria di Bologna, Via Albertoni-15, 40138 Bologna, Italy; 6 Department of Medicine and Surgery, University of Parma, 43121 Parma, Italy; 7 The Medical Oncology Centre of Rosebank, 129 Oxford Road, Saxonwold, Johannesburg 2196, South Africa; 8 Department of Immunology, Faculty of Health Sciences, University of Pretoria, Corner Doctor Savage Road and Bophelo Road, Pretoria 0002, South Africa; 9 Department of Medical Oncology, MD Anderson Cancer Center Madrid, 28033 Madrid, Spain

**Keywords:** circadian rhythm, clock genes, renal cell carcinoma, immunotherapy, HIF

## Abstract

**Simple Summary:**

Circadian rhythms are physical, mental, and behavioral changes that follow a 24-h cycle. These natural processes primarily respond to light and dark, and affect most living things, including animals, plants, and microbes. Circadian rhythm is also involved in the regulation of cellular differentiation and physiology as well as in the modulation of the immune system. Some genes controlling circadian rhythm may be implicated in the occurrence of common malignant cancers, including renal cell carcinoma. Recent studies showed that time-of-day infusion directly conditions the efficacy of immunotherapy in patients with cancer. Drugs targeting the circadian clock have been identified and their role in the era of immunotherapy should be investigated. In this review, we illustrate the role of clock genes in kidney cancer onset, progression and prognosis, and the potential therapeutic consequences of this emerging evidence.

**Abstract:**

Circadian rhythm regulates cellular differentiation and physiology and shapes the immune response. Altered expression of clock genes might lead to the onset of common malignant cancers, including Renal Cell Carcinoma (RCC). Data from Cancer Genome Atlas (TCGA) indicate that clock genes *PER1-3*, *CRY2*, *CLOCK*, *NR1D2* and *RORα* are overexpressed in RCC tissues and correlate with patients’ prognosis. The expression of clock genes could finely tune transcription factor activity in RCC and is associated with the extent of immune cell infiltration. The clock system interacts with hypoxia-induced factor-1α (HIF-1α) and regulates the circadian oscillation of mammalian target of rapamycin (mTOR) activity thereby conditioning the antitumor effect of mTOR inhibitors. The stimulation of natural killer (NK) cell activity exerted by the administration of interferon-α, a cornerstone of the first era of immunotherapy for RCC, relevantly varies according to circadian dosing time. Recent evidence demonstrated that time-of-day infusion directly affects the efficacy of immune checkpoint inhibitors in cancer patients. Compounds targeting the circadian clock have been identified and their role in the era of immunotherapy deserves to be further investigated. In this review, we aimed at addressing the impact of clock genes on the natural history of kidney cancer and their potential therapeutic implications.

## 1. Introduction

The circadian rhythm is a hierarchically cyclic system that regulates the daily oscillations of physiological processes and can respond to external environmental changes to maintain internal homeostasis [1]. Starting in the 1980s, studies culminating in the characterization of the first clock gene (CG) in *Drosophila melanogaster* paved the way for the characterization of additional genes and proteins, leading to what is presently known as the circadian clock (CC) [2].

CC genes control and sustain circadian rhythms in many pathophysiologic processes [3]. Disruptions in circadian rhythms are implicated in several pathologies such as diabetes, cardiometabolic and neurodegenerative disorders, and cancer. Circadian disturbance by shiftwork, jet lag, late night light exposure, and late-night food binging has been long linked to increased cancer risk. Furthermore, loss of circadian rhythmicity in patients has been associated with poor response to anti-cancer therapies and increased early mortality rates amongst cancer patients [4].

Renal Cell Carcinoma (RCC) is one of the most common urinary cancers worldwide, with a predicted increase in incidence in the coming years [5,6]. Partial or total nephrectomy is the gold standard curative treatment approach for patients with localized disease. Unfortunately, up to 30% of patients present with local or distant recurrence after surgery, thus requiring palliative systemic therapies [7,8]. From 2005–2015, tyrosine kinase inhibitors (TKIs) targeting vascular endothelial growth factor receptor (VEGFR) have represented the mainstay of metastatic renal cell carcinoma (mRCC) treatment [9]. Immunotherapy, as a single agent, in doublets, or in combination with anti-VEGFR TKIs, has rapidly become a cornerstone of the RCC therapeutic armamentarium since the approval of nivolumab in 2015, leading to a marked improvement in patients’ quality of life (QoL) and survival [10,11]. Although crucial advancements have been made to cure this disease, most mRCC patients have primary or acquired resistance to targeted therapy and immunotherapy, thus underlining the necessity of developing novel, personalized therapeutic approaches. 

In this review, we explored the role of clock genes in kidney cancer occurrence, progression, and prognosis and their potential therapeutic implications. 

## 2. Role of Clock Genes in Cancer

Mounting evidence supports the existence of molecular interconnections between CC and cancer. Many recognized cancer hallmarks such as copious metabolic demands, a favorable inflammatory microenvironment, immune suppression, and resistance to cell death, have a well-established circadian component. Hence, it might be conceivable that oncogenic transformation may lead to malfunctioning of the CC, which in turn creates a homeostatic imbalance, facilitating cancer growth and progression; on the other hand, it could be also speculated that CC malfunction could predispose to oncogenic transformation. 

The CC genes can be divided into two operation levels: systemic and cellular [11]. The central clock at a systemic scale, known as the “master” clock, is regulated by the central nervous system in the anterior hypothalamus [12] and is responsible for coordinating the cell-autonomous clocks in peripheral tissues and the brain in response to environmental stimuli [13]. At the cellular level, the CC genes are regulated by positive and negative transcription-translation loops which control the rhythmicity of cellular, metabolic, and physiologic events [14]. At the transcriptional level, the clock is driven by positive loop regulators: basic helix–loop–helix heterodimeric transcription factors regulate the expression of key circadian genes, which are the negative regulators of the circadian loop. Modifications of some circadian genes at the translational level regulate protein stability, control nuclear entry of repressor protein complexes, and impact the clock’s autoregulatory feedback loops [14]. 

There are eight core CC genes involved with the circadian rhythm: Period1 (*PER1*), period2 (*PER2*), period3 (*PER3*), cryptochrome1 (*CRY1*), cryptochrome2 (*CRY2*), aryl hydrocarbon receptor nuclear translocator-like protein 1 (*BMAL1*), neuronal PAS domain protein 2 (*NPAS2*), and circadian locomotor output cycles protein kaput (*CLOCK*). The *PER1*, *PER2,* and *PER3* genes regulate cell growth, proliferation, and apoptosis [15,16], *CRY1* and *CRY2* regulate the transcription G1/S and G2/M cell cycle checkpoints [17], while *BMAL1* and *NPAS2* inhibit the proliferation and invasion of cancer cells by suppressing the c-Myc transcription factor [18]. The *CLOCK* gene enhances VEGF-mediated angiogenesis in cancer cells and metastatic invasion by interacting with HIF-1α/BMAL1 [19]. Interestingly, down-regulation of *PER1-3*, *CRY2*, *NPAS2*, *BMAL1,* as well as *CLOCK* gene expression, correlates with high histological grade and poor prognosis [20] and short survival in different cancers [21,22]. 

The epithelial-to-mesenchymal transition (EMT) is a crucial step in cancer progression and enables cancer cell metastasis. Low expression of *PER2* led to the activation of EMT genes *TWIST1* and *SLUG* and promoted cancer metastasis [23]. In addition, some components of the CC have a significant antitumor effect through cell cycle arrest, the DNA damage response, and correlation with essential pathways, including the p53 [24]. 

Investigations are currently ongoing to evaluate other CC genes, such as casein kinase 1ε (*CK1ε*), receptor subfamily 1 group D member 1/2 (*NR1D1/2*), RAR-related orphan receptor α and β (*RORα/β*), timeless (*TIMELESS*) and timeless-interacting protein (*TIPIN*). To date, although far more aware of their essential role in feedback loop regulation, data on the real contribution of these CC genes in cancer development and progression are still not sufficient or conclusive [25].

## 3. Role of Circadian Clock Genes in Renal Cell Carcinoma Tumorigenesis and Prognosis

Animal models of genetic disruption of CC genes have been strongly associated with different cancers, including RCC, prostate, breast, colon, liver, pancreas, ovary, and lung malignancies [26]. In Wilms tumors, rare kidney cancers that primarily affects children, the expression of CLOCK protein is dramatically reduced in tumor cells, suggesting that the CC molecular axis may be disrupted in dedifferentiation-mediated embryonal tumors [27].

**Figure 1:**
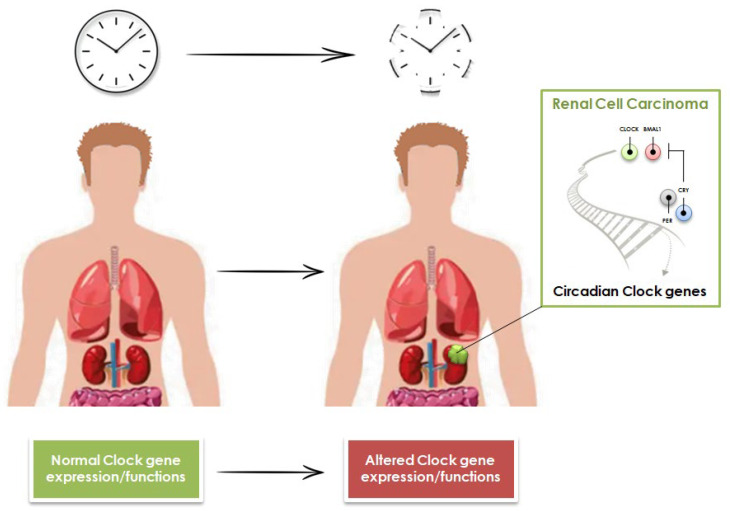
Role of Circadian Clock genes in the onset of Renal Cell Carcinoma.

In RCC, the CC circuitry is deregulated, and the altered expression of CC genes might contribute to tumor onset and progression (Figure 1). 

Mazzocoli et al. [28] evaluated the expression of CC genes by DNA microarray assays and qRT-PCR in 11 RCC primary tumors and matched healthy tissues. They documented down-regulation of *PER2*, *TIMELESS* and *TIPIN* and up-regulation of *SERPINE1* genes. Furthermore, a statistically significant correlation between mRNA levels of *PER2* and *CSNKIE*, *PER2* and *TIPIN*, *PER2* and *SERPINE1*, *TIMELESS* and *TIPIN*, *TIMELESS* and *CSNKIE*, *TIPIN* and *CSNKIE* was reported [28]. 

**Figure 2:**
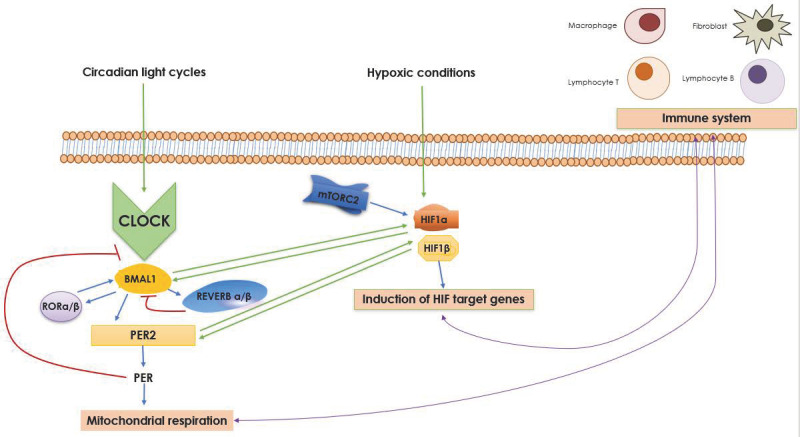
Relationship between HIF and CLOCK pathway. Under circadian light cycles, CLOK protein combines with BMAL1 protein to generate BMAL1/CLOCK heterodimer, which cause transcriptional activation of core clock genes (for example, transcription of *PER2*); this in turn inhibits BMAL1/CLOCK dimer activity. Meanwhile, the BMAL1/CLOCK dimers activate the transcription of the *Rev-Erb-α/β* and *ROR*α/β genes, and the resulting translated proteins inhibit and promote the transcription of BMAL1, respectively. PER pathway regulates many transcriptional–translational processes influencing the whole cell metabolism and particularly mitochondrial activity. Together with the mTOR axis, hypoxia inducible factor (HIF) pathway orchestrates responses to oxygen and nutrient availability. HIF-1α can increase the amplitude of the PER2 circadian rhythm oscillation; colocalize with BMAL1 on E-box regions to increase expression of circadian and HIF target genes; CLOCK and BMAL1 can also express the *HIF-1α* gene. HIF patwhay activation and mitochondrial respiration can interplay with immune response. BMAL1 = Brain and Muscle ARNT-Like 1; REVERB α/β (REV-ERB-α and REV-ERB-β) = nuclear receptors that regulate the expression of genes involved in the control of circadian rhythm, metabolism and inflammatory responses; RORα/β = RAR-related orphan recep-tor alpha/beta; CLOCK = circadian locomotor output cycles protein kaput; PER2 = period2; PER = period; mTORC2 = mammalian target of rapamycin complex-2; HIF1α and HIFβ = hypoxia inducible factor-α and hypoxia inducible factor-β.

CC system also shapes the activity of the mammalian target of rapamycin (mTOR) pathway, which is crucial for the development of RCC (Figure 2) [29]. 

The expression levels of mTOR proteins show a 24-h rhythm in RCC tissues, mainly due to the activity of the ubiquitination factor Fbxw7, which is regulated by the circadian regulator D-site-binding protein. Of note is the fact that, the administration of everolimus, an oral mTOR inhibitor, improved survival in animal models during periods of elevated mTOR expression [30].

Together with the mTOR pathway, hypoxia inducible factor (HIF) pathway orchestrates responses to oxygen and nutrient availability. HIF plays a vital role in renal tumorigenesis, being constitutively activated by inactivation of the von Hippel-Lindau gene. Recently, the role of HIF as a potential therapeutic target in RCC patients has been supported by the positive results obtained by Belzutifan (MK-6482), a potent selective small molecule inhibitor of the HIF-2α subunit [31]. In Caki-2 RCC cell lines, an interaction between PER2 and HIF has been observed. Indeed, it has been shown that HIF-1α can increase the amplitude of the PER2 circadian rhythm oscillation by directly binding to the HIF-binding site located on the PER2 promoter [32]. 

As for the prognostic role of CC genes, the analysis of Cancer Genome Atlas (TCGA) data reported that the overexpression of *PER1-3*, *CRY2*, *CLOCK*, *NR1D2,* and *RORα,* as well as the under-expression of *TIMELESS* and *NPAS2,* were correlated with longer survival in patients with RCC [33]. Furthermore, the expression of *PER2*, *DBP*, *PER3*, *CRY2*, and *RORα,* has been shown to be significantly associated with kidney cancer prognosis [34]. Interestingly, in this study the expression of *PER2*, *DBP*, *PER3*, *CRY2*, and *RORα* genes was positively associated with the infiltration levels of CD4 and CD8 T cells [34].

## 4. Circadian Variations of Cytokines and Chemokines Involved in Renal Cell Carcinoma

Pro-inflammatory cytokines, such as Interleukin (IL)-1, IL-6 and tumor-necrosis factor (TNF)-α, and the expression of inflammatory chemokines (i.e., CXCL9 and CXCL10) has been associated with RCC tumor growth, angiogenesis, and response to therapy [35,36,37]. On the other hand, anti-inflammatory cytokines, including IL-10 and transforming growth factor (TGF)-β, and non-inflammatory chemokines (i.e., CCL17 and CCL24) produced by M2 phenotype tumor-associated macrophages (TAMs) promote tissue remodeling and angiogenesis in RCC microenvironment [38].

Moreover, the levels of several cytokines and chemokines involved in RCC onset and progression vary during the 24 h day. In this regard, it has been observed that the secretion of IL-6 varies during daytime, with two nadirs at about 8.00 and 21.00. Of note, IL-6 secretion is strictly associated with the sleep-wake rhythm and results high in disorders of excessive daytime sleepiness such as narcolepsy and obstructive sleep apnea [39].

Furthermore, TNF-α and TGF-β have been shown to regulate the transcription of the CC genes [40]. In turn, circadian oscillations of TNF-α gene expression are regulated by clock genes *BMAL1* and *CLOCK1* [41].

As for chemokines, the *PER2* gene regulates the levels of CCL5 (Rantes), a C-C chemokine secreted by T lymphocytes late after activation, fibroblasts, epithelial cells, and endothelial cells after stimulation by TNF –α and interleukin-1β [42]. 

## 5. Evidence on the Time-of-Day Influence on the Efficacy of Immunotherapy and Targeted Therapy

The time-of-day of treatment administration has emerged as a potential factor on the effectiveness of anticancer drugs. These chrono-pharmacological phenomena result of bot from the pharmacodynamics and pharmacokinetics of these agents, which are affected by the activity of CC genes. In this regard, Hori et al. reported that the delivery of anti-cancer drugs to tumor tissues varies following the circadian oscillations of blood flow in tumor tissues [43]. Similarly, studies in mice reported reduced tumor growth when cyclin-dependent kinase 4/6 (CDK4/6) drugs were administered in a time-dependent manner, showing higher efficacy in a morning dosing regimen compared to nighttime dosing [44]. 

Chronotherapy is defined as the administration of a treatment in coordination with the body’s circadian rhythms; the aims of chronotherapy were the optimization of efficacy and minimization of adverse events. In the study led by Deprés-Brummer et al. in 1991 [45], ten patients with advanced RCC or melanoma were treated with 15–20 MU/m^2^/day recombinant alpha-interferon-2b through a continuous 21-day intravenous schedule at circadian modulated rate. Compared with standard administration schedules, this circadian infusion schedule registered a significant increment in the total daily dose and dose intensity, with seven of the ten patients alive at a median follow-up of 15 months and two patients who continued chronotherapy for at least 9 and 13 months, respectively. 

In 2009, Shiba et al. [46] investigated the expression of type-1 interferon receptor (IFNAR2) in peripheral blood mononuclear cells (PBMC) from RCC patients highlighting a peak at night, followed by a downregulation within 48 h from IFN-alpha administration and a subsequent recovery within further 48 h. These findings were in line with the results published in 1995 on the seven-day continuous infusion of IFN-α through a circadian schedule with maximum delivery between 6 p.m. and 3 a.m. [47].

Immunotherapies targeting immune checkpoint receptors or ligands such as Cytotoxic T-Lymphocyte-Associated protein-4 (CTLA-4), Programmed Death -1 (PD-1) and its ligand (PD-L1), are currently being widely exploited in clinical trials for multiple cancer types. Nonetheless, these agents are known to induce a significant inflammatory response and immune-related adverse events [48]. Since immune cell trafficking and inflammatory pathways are under CC control, applying a chronotherapy approach could help mitigate the associated toxicity issues [49]. The influence of time-of-day infusion on the effectiveness of immune checkpoint inhibitors has been recently investigated by Qian et al. in patients with advanced melanoma treated by ipilimumab, nivolumab, or pembrolizumab [50]. Specifically, patients 20% who had received at least of infusions after 4.30 pm (the cut off time delineating the onset of the evening) reported the worst overall survival (OS) [Hazard Ratio (HR) 1.31, 95% Confidence Interval (CI) 1.00–1.71, *p =* 0.046]. Accordingly, at a propensity score-matched analysis, median OS was shorter in patients who received at least 20% of immunotherapy infusions after 4.30 pm (4.8 years vs. not reached, HR 2.04, 95%CI: 1.08–2.98, *p* = 0.023) [50,51]. 

More recently, our group had observed that patients treated by dual immune checkpoint inhibitors or by the combination of immune checkpoint inhibitors with anti-VEGR TKIs before 4.30 pm had a significantly longer median PFS compared with those with the latest administration (12.3 vs. 5.6 months; HR 2.28: 95%CI 1.1–5.15; *p* = 0.048). Overall Survival data were not mature but demonstrated a trend toward improved survival for patients with earlier infusion administration (HR 2.33 *p* = 0.16) [52]. 

## 6. Emerging Strategies to Modulate Clock Genes in Patients with Cancer

Compounds targeting the CC have been developed as potential therapies for clock-related diseases, including cancer. Oshima et al. [53] identified by phosphoproteomics a potent and selective inhibitor of CK2 named GO289. This molecule can inhibit multiple phosphorylation sites on clock proteins, including PER2 S693, leading to decreased cancer cell growth. More recently, Borgo et al. [54] compared the efficacy and selectivity of CK2 inhibitors through a phosphoproteomics approach, concluding that both GO289 and CX4945 showed negligible off-target effects and high inhibitory efficacy against CK2. 

Currently, an observational study (COMBOREIN, NCT03571438) investigating the treatment of RCC patients’ cell cultures with the combination of CK2, and ATM inhibitors (compared to sunitinib, pazopanib, or temsirolimus) is ongoing, with the study completion date estimated at 30 September 2024. 

Promising strategies to modulate clock proteins in patients with cancer consist of small molecules targeting biological clock, and synthetic anticancer chronobiotics directed against mammalian circadian clock components (i.e., CRYs, REV-ERBs, and RORs), as well as casein kinases [55].

## 7. Discussion

It is well known that CC genes and the cell cycle are tightly coupled, cooperating for proper cell functioning, and the dysregulation of the CC can significantly affect cell homeostasis and promote cancer development [3]. While the functions of the CC genes in normal physiology have been fully elucidated, studies of CC gene alterations in cancer are still lacking, leaving a gap in the clarity and description of their functions in neoplastic cells. 

In RCC, the lack of validated biomarkers of efficacy or resistance of current therapeutic approaches strongly supports the necessity of exploring a spectrum of clinical and behavioral variables that could impact on patient outcome. Diet [56,57], fasting [58], concomitant medications [59,60,61,62], physical activity [63], and various other factors have been linked to immunotherapy efficacy in RCC. In this scenario, dosing time is an emerging element to be considered while managing RCC patients.

Including chronotherapy in daily therapy for RCC may offer a more effective and less toxic approach, although biomarkers for chronotherapy strategies’ efficiency still need to be adequately defined [64]. Nevertheless, the results on the timing of immune checkpoint administration in patients with melanoma and RCC clearly indicate the clinical relevance of chronotherapy.

Well-designed, larger-size, and higher-quality cancer patient cohort studies are needed to investigate the precise impact of CC genes on the pathobiological behaviors of cancers. Additional in vitro and in vivo experiments and ad hoc clinical trials are warranted to better elucidate CC involvement in RCC biology, with the ultimate aim to improve patient prognosis and QoL. Finally, we need to plan dedicated clinical trials to assess: (1) the combination of emerging drugs regulating the CC with current standards; (2) the employment of CC targeting agents as maintenance treatment, (3) and as adjuvant treatment in patients with high-risk of relapse. In particular for mRCC, we could hypothesize to design a randomized trial aimed to assess the impact of treatment administration before and after 4.30 p.m. 

## 8. Conclusions

Role of CC genes and circadian rhythm in patients with RCC is an intriguing and recent area of research, particularly considering the new findings on the impact of circadian rhythm in anticancer therapy. To date, evidence clearly supports the idea that CC genes and proteins may represent future therapeutic targets and the time of administration of immunotherapy drugs during the day is a nonnegligible factor.
